# Financial Symmetry and Moods in the Market

**DOI:** 10.1371/journal.pone.0118224

**Published:** 2015-04-09

**Authors:** Roberto Savona, Maxence Soumare, Jørgen Vitting Andersen

**Affiliations:** 1 Department of Economics and Management, University of Brescia, Brescia, Italy; 2 Laboratoire J.-A. Dieudonné, Université de Nice-Sophia Antipolis, Nice, France; 3 CNRS, Centre d’Economie de la Sorbonne, Université Paris 1 Pantheon-Sorbonne, Paris, France; University College London, UNITED KINGDOM

## Abstract

This paper studies how certain speculative transitions in financial markets can be ascribed to a symmetry break that happens in the collective decision making. Investors are assumed to be bounded rational, using a limited set of information including past price history and expectation on future dividends. Investment strategies are dynamically changed based on realized returns within a game theoretical scheme with Nash equilibria. In such a setting, markets behave as complex systems whose payoff reflect an intrinsic financial symmetry that guarantees equilibrium in price dynamics (fundamentalist state) until the symmetry is broken leading to bubble or anti-bubble scenarios (speculative state). We model such two-phase transition in a micro-to-macro scheme through a Ginzburg-Landau-based power expansion leading to a market temperature parameter which modulates the state transitions in the market. Via simulations we prove that transitions in the market price dynamics can be phenomenologically explained by the number of traders, the number of strategies and amount of information used by agents, all included in our market temperature parameter.

## Introduction

**Fig 1:**
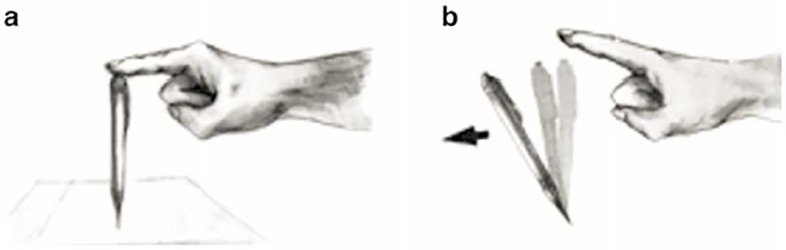
A pen falls and a symmetry is broken. The Fig. 1A depicts symmetric state of a pen with no clear direction taken. The state is symmetric since there is no a priori preferred direction in which the pen would fall. Fig. 1B describes the pen falling in one specific direction corresponding to a symmetry break when the finger is lifted. The symmetry break happens because a small fluctuation in the positioning of the pen inevitable happens when lifting the finger, leading to a change of the system from one state (pen held upright on a table with a finger) to another (pen suddenly falls in an arbitrary direction).

Think of a pen held upright on a table with a finger, and imagine to slowly lifting your finger until the pen suddenly falls in an arbitrary direction as depicted in Fig. 1A-B. What has this in common with financial market dynamics? Both are systems in which whenever a small fluctuation makes the system cross a critical point, the system moves into one or more definite states. The phenomenon where a system goes from a symmetric but disordered or random state (the pen can fall into any given direction) into an ordered state in which the symmetry is broken and the pattern is well defined (the pen is falling in one specific direction) is what characterizes a phase transition in physics. In this paper we develop a theoretical framework for financial markets based on this phenomenological reasoning: the pen in the symmetric state is equivalent to price fluctuations around their fundamental value, whereas the pen falling (the symmetry is broken) is equivalent to price trends towards herding seen in a bubble phase or anti-bubble phase of the market.

The key question we face is to explain when and why financial symmetry breaking is taking place, corresponding to the financial system moving from one state (or mood), where randomness in price movements obey no-arbitrage conditions, into another state in which the price takes the route towards bubble or anti-bubble formation giving rise to arbitrage conditions. The first mood is the *fundamental state*, while the second is the *speculative state*.

A large body of literature has explored markets seen as complex systems with bounded rational interacting agents. A possible, while non-exhaustive list of references includes Refs. [1–9]. The common feature of these studies concerns the way they model dynamical interactions of agents’ choice, which are stylized within a ‘mean-field’ approach where the intensity of choice is equivalent to a temperature. In more depth, complex systems are assumed as “… dissipative structures that import free energy and export entropy in a way that enables them to self-organize their structural content and configuration, subject to boundary limits” Ref. [8]. In such a reasoning, markets are assumed as thermodynamically systems where energy (order) and entropy (disorder) are struggling against each other moving the markets from one state to another based on their inner “temperature”.

In our paper we take the same “anti-reductionism” (or “metaphysical”) perspective, where financial systems are not merely the sum of single parts but, instead, they reflect very complex dynamics that can be understood only by inspecting the system as a whole, enweaving micro-to-macro connections within a consistent economic framework that takes into account linear and non-linear interactions. Instead, conventional economic theory is often unable to describe such dynamics being based on a “reductionist” approach, in which the micro-economic behavior of a representative agent is first stylized using rational, bounded rational or behavioral frameworks, then next used in a synthesis of macro behaviors. In other terms, the state (or mood) of the system is the macroscopic result of many microscopic decisions.

The “dissipative” financial market structure we have in mind is characterized by two macro-configurations shaped by the spatiotemporal interactions of single agents: (1) a *speculative state*, in which single traders take the same direction (all buy or sell), leading to market trends of bubbles and anti-bubbles; (2) a *fundamental state*, where traders put different buy and sell orders leading the price path to move around its fundamental value.

Agents are assumed to be bounded rational, using a limited set of information including past price history (technical analysts) and expectation on future dividends (fundamentalists). Investment strategies are dynamically changed based on realized returns within a game theoretical scheme with Nash equilibria. In such a setting, the market payoff reflects an intrinsic financial symmetry that guarantees invariance inside the system until the symmetry is broken, then leading the market towards speculative scenarios. State transitions appear whenever the “temperature” of the market crosses a critical transition point, which marks the speculative vs. fundamental behaviors.

Our main innovation is to give a formal, theoretical-based measure to such a market temperature.

The collective “choice” of the system which we refer to as “aggregate decision making” is conducive to answer how prices are formed over time and describes states of the market, which Rational Expectations Hypothesis (REH) or even the bounded REH cannot address. Indeed, once asset prices and dividends are both included in the decision making process, the REH suggests agents should make their trading decisions based on dividends only, but the matter becomes far from trivial if you try to explain generally what will be the collective outcome of *N* people’s decision making. Are decisions based on the price of the asset in anticipation of future price behavior (speculative state), or are rather the anticipation of future dividends the only driver of agents’ trading decisions (fundamentalist state)?

Our framework is able to give an answer to this question by coarse-graining the agents’ interactions through the well-known Ginzburg-Landau (GL) theory in physics, used to describe superconducting transition in terms of a complex order parameter field. The adaptation of the GL theory to our problem allows us to bypass the excessively complicated microscopic description of moods in the markets, by means of an “order parameter” that modulates transitions between fundamental and speculative states without examining the micro-dynamics of single agents and their interaction impacts on the price path and connected market transitions.

We proceed as follows. We first describe the multi-agent-based model used to describe the payoff functions of single agents and related impacts on market returns. Then we describe the rationale of market phase transitions through the GL theory, next reporting and commenting results from simulation study. We conclude summarizing our main contributions also outlining our future research agenda.

## Multi-Agent Based Modeling

The theoretical framework used to formalize the speculative and the fundamental states refers to the “$-Game” multi-agent based modeling approach proposed in Ref. [10], in which single agents make their investment decisions based on dividends and asset prices with the objective to maximize their profit payoff function.

The $-Game is an extension of the Minority Game (MG), introduced in Ref. [11] and implemented in many studies on market price dynamics Refs. [12–14]. The basic MG scheme consists in a repeated game where the players choose 1 out of 2 alternatives (buy or sell) at each time step based on past information, and the winning agents are those in the minority group. Such a scheme was introduced following the leading principle in Physics for which, in order to solve a complex problem one should first identify essential factors at the expense of trying to describe all aspects in detail.

Similarly, the $-Game is our “minimal” model to describe and predict financial market dynamics. Although simple in principle, the $-Game yields rich system dynamics, the complexity of which can be acted upon by the choice of system parameters (memory length, liquidity, etc.). As will be shown below, this thereby creates a dilemma in terms of the investment strategies of the participants, and the pure cases of speculative vs. fundamentalist states will appear as special cases of the general theory. Differently from MG, in the $-Game the best strategies are not always targeting the minority but are shifting opportunistically between the minority and the majority.

The mathematical definition of the model includes *N* players (or agents) that simultaneously take part in a one-asset financial market over a time horizon of T periods. At each *t* period, with t < T, each player *i* chooses an action *a*_*i*_(*t*) ∈ {−1, 1}, where the action −1 is a “sell” order and the action 1 is a ”buy” order. In submitting buy and sell orders, agents can use: (1) technical analysis strategies, trying to profit from *past* price trends, and (2) fundamental analysis strategies, based on expectation of *future* dividends. The aggregate choices that look at the past lead to a *pure speculative state* (technical analysis strategies), and the aggregate choices that look at the future lead to a *pure fundamental state* (fundamental analysis strategies). Players are assumed to be bounded rational, in the sense of using only a limited informational set (past price trends and dividends) to make their decisions, with no short-sales constraints.

### Speculators

In the speculative state a majority of the agents are technical analysts (chartists) who only analyze past realization of prices, with no anchor on fundamental economic analysis. Each player observes the history of past *m* ∈ ℕ price movements in making decision of whether to buy or sell an asset. Therefore, *m* denotes the size of the agent’s memory or, putting in other terms, the length of the signal used in the decision making process. To take decisions the players have at their disposal a fixed number of *s* strategies, which are by construction randomly assigned at the beginning of the game. Thus, a specific strategy *j* tells whether to buy or sell an equity depending on the past price history of up and down moves, represented as 1 (up) and 0 (down), respectively.

At each time *t* the *i*-th player uses his/her *best* (in terms of payoff see Eq. 1 below) strategy taking an action $a_{i}^{*}\left(t\right)$ of either buying $a_{i}^{*}\left(t\right)=+1$ or selling $a_{i}^{*}\left(t\right)=-1$ (the * is used to denote it is the best strategy). It follows that in general a given strategy $a_{i}^{j}$ is a mapping from the set of histories of size *m* to {−1, +1}.

Table 1 shows an example of a given strategy for *m = 3*. For all possible histories of up and down market moves over the last *m* time steps, the strategy suggests a specific action to take at time *t*, namely $a_{i}^{j}\left(\vec{h}\left(t\right)\right)=\pm 1$ with$\vec{h}\left(t\right)\in {\left\{0,1\right\}}^{m}$. For instance, if the market went down over the last m = 3 days, the strategy in Table 1 suggests to buy the stock (000→ +1). If instead the market went down over the last two days and then up today, the same strategy suggests to sell (001→-1). Note again in Table 1 that, since the price has 2 possible moves (up or down) we have a space of possible past paths equal to 2^*m*^ = *8* bit sequences of 0’s and 1’s each one corresponding to a specific action {−1, +1} suggested by the *j* strategy. This signifies that the space of all possible strategies is given by$2^{2^{m}}=256$ alternatives corresponding to each of the 8 bit sequences.

**Table 1:** Example of speculative trading strategy.

History $\vec{h}\left(t\right)$	Action $a_{i}^{j}(t)$
000	1
001	−1
010	−1
011	1
100	−1
101	−1
110	1
111	−1

The table shows an example of a technical trading strategy for an investor with a history of past price movements of *m* = 3 time steps. For all possible histories of up and down market moves over the last *m* steps, the strategy suggests a specific action to take at time *t*, namely buy ($a_{i}^{j}\left(t\right)=1$) or sell ($a_{i}^{j}\left(t\right)=-1$).

While a single strategy recommends an action for all possible histories (of length *m*), we also allow for agents to adopt different strategies over time. Namely, agents keep a record of the overall payoff each strategy would have yielded over the entire market history (i.e. not limited to *m* past periods) using a rolling window of size m, and use this record to update which strategy is the most profitable see Eq. (1) below. This renders the game highly non-linear: as the price behavior of the market changes, the best strategy of a given agent changes, which then can lead to new changes in the price dynamics.

The action corresponding to the best strategy taken by agent *i* at time *t* is denoted by$a_{i}^{*}\left(t\right)=\pm 1$, while ${\left(a_{i}^{*}\left(t\right)\right)}_{i}\in {\left\{-1,+1\right\}}^{N\times T}$ is the *action profile* of the entire market population, where ${\vec{a}}^{*}\left(t\right)={\left(a_{1}^{*}\left(t\right),\ldots ,a_{N}^{*}\left(t\right)\right)}_{i}\in {\left\{-1,+1\right\}}^{N}$ corresponds to the action played by the *N* agents in period *t*.

The payoff π of the *i*-th agent’s *j*-th strategy, $a_{i}^{j}\left(t\right)$, in period *t* is determined as follows:

$$\pi \left[a_{i}^{j}\left(t\right)\right]=a_{i}^{j}\left(t-1\right)r\left(t\right)$$ (1)

In Eq. (1) *r(t)* denotes the return of the market between time *t*−1 and *t*. The payoff in the $-Game therefore describes the gain/loss obtained by an investment strategy executed at time *t−*1 depending on the market return in the following time step *t*. I.e. $-Game agents are investors that try to predict and profit from future market movements. *r(t)* can in turn be expressed in terms of the global order imbalance, $\sum_{k=1}^{N}a_{k}^{*}\left(t\right)$, divided by the liquidity of the market λ, as discussed below.

As proven in Refs. [15, 16] traders’ actions impact significantly on price returns and liquidity through a positive autocorrelation in equilibrium imbalances reflected in a positive predictive relation between imbalances and future returns. Price return *r(t)* from *t−*1 to *t* can be then assumed as proportional to the order imbalance, leading to the following

$$r\left(t\right)\equiv \text{ ln}p\left(t\right)-\text{ln}p\left(t-1\right)=\lambda^{-1}\sum_{k=1}^{N}a_{k}^{*}\left(t\right)$$ (2)

with *p*(∙) denoting the price of the stock and λ is a parameter describing the liquidity of the market with λ ∝ *N*. Note that the price goes in the direction of the sign of the order imbalance according to Refs. [17, 18].

Therefore the payoff function in Eq. (1) can be re-expressed in terms of the return function in Eq. (2) as:

$$\pi \left[a_{i}^{j}\left(t\right)\right]=a_{i}^{j}\left(t-1\right)\sum_{k=1}^{N}a_{k}^{*}\left(t\right)/\lambda$$ (3)

To summarize: in the $-Game technical analysis trading strategies are based on a rewarding scheme for strategies that at time *t—*1 predicted the proper direction of the return of the market *r*(*t*) in the next time step *t*. The larger the move of the market, the larger the gain/loss depending on whether the strategy properly/improperly predicted the market move. If the agent correctly anticipates the right direction of the market, the profit will be positive and equal *to N/λ* in both the extremes where either all agents sell or buy [-*N*; +*N*], having in fact (-1 ×—*N* ≡ *+1 × +N = N)/λ* from Eq. (2).

### Fundamentalists

Differently from pure speculators, fundamentalist make their investment decisions based on fundamental economic analysis. Each player makes forecasts on future price based on expected dividends *D(t)* over the entire time horizon, thereby obtaining a fundamental price *p*_*f*_(*t*) estimation equivalent to the expectation of all future dividends discounted at a constant risk-free rate *ρ*, at which investors can buy or sell the stock:

$$p_{f}(t)=E_{t}[\int_{t}^{T}e^{-\rho (\tau -t)}D(\tau )d\tau ]$$ (4)

with *E*_t_[*D*(τ)] = *D*(t) which implies in the continuum a variation in *p*_*f*_ from *t* to *t* + *dt* as a martingale with σ constant and *dZ* denoting the standard Brownian motion:

$$dp_{f}\left(t\right)=\sigma dZ$$ (5)

As result, a change in the fundamental price reflects the arrival of news regarding future cash flows of the stock.

Buy and sell actions made by fundamentalists at time *t*, $a_{i}^{f}\left(t\right)$, are based on the value of *p*(t) vs. *p*_*f*_ *(t)* reflecting the common rule of thumb to sell when price is high (higher than the fundamental), and buy when price is low (lower than the fundamental):

$$a_{i}^{f}\left(t\right)=\left\{ \begin{array}{l} -1\;if\;p\left(t\right)>p_{f}\left(t\right)\\ +1\;if\;p\left(t\right)<p_{f}\left(t\right) \end{array}\right.$$ (6)

Furthermore, in order to limit the sell orders whenever *p*(t) ≫ *p*_*f*_ *(t)*, which would rather indicate a speculative scenario[22], we assume a vanishing use of the fundamental strategy according to a Poisson process of the form γ*e* ^−γ^ with $\gamma =\frac{p\left(t\right)-p_{f}\left(t\right)}{D\left(t\right)}$. This assumption provides a maximum probability in choosing the fundamental strategy in correspondence with a price variation from its fundamental almost equivalent to the dividend yield, which makes sense given that the fundamental price is the expectation of future dividends.

The payoff function for fundamentalists is the same as that of speculators (see Eqs. (1)–(3)), the only change is the action selection mechanism given by (Eq. 6).

### Fine-grained market dynamics

The formulation of the decision making process of speculators and fundamentalists via the $-Game leads to a financial market modeling based on 5 parameters:

- *N*—The number of market participants which expresses the “physical” size of the market.
- *m*—The memory length of the signal used by the agents in their decision making process when they act as speculators. This is expressed in terms of the past number of days the agents look at when they decide whether to buy or sell an asset.
- *s*—The number of strategies held by the agents when they act as speculators. By construction the *s* strategies of each agent is chosen randomly in the total pool of $2^{2^{m}}$ strategies at the beginning of the game.
- λ—The liquidity parameter of the market. λ will be assumed proportional to *N*
- *D*(*t*)—The future expectations about the dividends paid over the entire time horizon which is assumed to be constant in time *t* for simplicity, hence D(t) ≡ D.

These 5 parameters are mixed together in a reflexive, non-linear $-Game-based market dynamics where each agent uses his/her best strategy at every time step giving rise to a complex system dynamics where, as the market changes, the best strategies of the agents change too, and as the strategies of the agents change, they thereby change the market.

The dynamics of the $-Game is driven by a nonlinear feedback mechanism because each agent used his/her *best* strategy (fundamental/technical analysis) at each time step. The sign of the order imbalance, $\sum_{i=1}^{N}a_{i}^{*}\left(\vec{h}\left(t\right)\right)$, in turn determines the value of the last bit *b(t*) at time *t* for the price movement history$\vec{h}\left(t+1\right)=\left(b\left(t-m+1\right),b\left(t-m\right),\ldots ,b\left(t\right)\right)$. The dynamics of the $-Game can then be expressed in terms of an equation that describes the dynamics of *b(t)* as:

$$b\left(t+1\right)=\Theta [\sum_{i=1}^{N}a_{i}^{*}\left(h\left(t\right)\right)]$$ (7)

where Θ is a Heaviside function taking the value 1 whenever its argument is larger than 0 and otherwise 0, and $h\left(t\right)=\sum_{j=1}^{m}b\left(t-j+1\right)2^{j-1}$ is now expressed as a scalar instead of a vector. The nonlinearity of the game can be formally seen from:

$$a_{i}^{*}\left(h\left(t\right)\right)=a_{i}^{\left\{j|{\text{max}}_{j=1,\ldots ,s}\left[\prod \left\{a_{i}^{j}\left(h\left(t\right)\right)\right\}\right]\right\}}\left(h\left(t\right)\right)$$ (8)

with

$$\prod \left\{a_{i}^{j}\left(h\left(t\right)\right)\right\}=\sum_{k=1}^{t}a_{i}^{j}\left(h\left(k-1\right)\right)\sum_{i=1}^{N}a_{i}^{*}\left(h\left(k\right)\right)$$ (9)

Inserting the Eqs. (9) and (10) in Eq. (8) one obtains an expression that describes the $-Game in terms of just one single equation for *b*(*t*) depending on the values of the 5 base parameters variables (*m;* s; *N*; λ; *D*(*t*)) and the random variables $a_{i}^{j}$ (i.e. their initial random assignments).

A major complication in the study of this system of equations happens because of the non-linearity in the selection of the best strategy, and the higher the number of *s* the complexity of the system gets bigger and bigger. The case of s = 2 is however simple to deal with, since one only need to know the relative payoff $q_{i}\equiv \pi \left[a_{i}^{1}\right]-\pi \left[a_{i}^{2}\right]$ between the two strategies Refs. [19–20]. Indeed, as proven in following proof for this special case the $-Game is in Nash equilibrium with only technical analysis strategies (with no cash nor asset constraints) akin to that of Keynes’ “Beauty Contest”, where it becomes profitable for the subjects to guess the actions of the other participants. The optimal state is then one for which all subjects cooperate and take the same decision (either buy/sell) leading the price into a bubble state where it deviates exponentially in time from its fundamental. All subjects profit from further price increases/decreases in the bubble state, but it requires coordination among the subjects to enter and stay in such a state, see the following proof:

We would first like to point out an important difference compared to traditional game theory since in our game the agents have *no* direct information of the action of the other players. The only (indirect) information a given agent have of other agent’s action through the aggregate actions of the past, i.e. the past price behavior. Let the action of optimal strategy $a_{i}^{*}$ be expressed in terms of the relative payoff, *q*_*i*_, so as to formulate $\sum_{i=1}^{N}a_{i}^{*}\left(h\left(t\right)\right)$ as follows

$$\sum_{i=1}^{N}a_{i}^{*}\left(h\left(t\right)\right)=\sum_{i=1}^{N}\left\{\Theta \left(q_{i}\left(h\left(t\right)\right)\right)a_{i}^{2}\left(h\left(t\right)\right)+\left[1-\Theta \left(q_{i}\left(h\left(t\right)\right)\right)a_{i}^{1}\left(h\left(t\right)\right)\right]\right\}$$ (10)

Inserting Eq. (10) into Eq. (7) and take the derivative of *b* in t + 1

$${\frac{db}{dt}|}_{t+1}=\delta \left(\sum_{i=1}^{N}a_{i}^{*}\left(h\left(t\right)\right)\right)\sum_{i=1}^{N}\{\delta \left(q_{i}\left(h\left(t\right)\right)\right)\frac{\delta q_{i}\left(h\left(t\right)\right)}{\delta t}\left[a_{i}^{2}\left(h\left(t\right)\right)-a_{i}^{1}\left(h\left(t\right)\right)\right]+\Theta \left(q_{i}\left(h\left(t\right)\right)\right)\frac{\delta a_{i}^{2}\left(h\left(t\right)\right)}{\delta t}+\left[1-\Theta \left(q_{i}\left(h\left(t\right)\right)\right)\right]\frac{\delta a_{i}^{1}\left(h\left(t\right)\right)}{\delta t}\}$$ (11)

Looking inside the bracket of the sum in (11), it follows that a change in $\sum_{i=1}^{N}a_{i}^{*}\left(h\left(t\right)\right)$ can occur either because the optimal strategy changes and the two strategies for a given *h*(*t*), $a_{i}^{1}\left(h\left(t\right)\right)$ and $a_{i}^{2}\left(h\left(t\right)\right)$, differ one each other (first term in the bracket). Furthermore, a change in $\sum_{i=1}^{N}a_{i}^{*}\left(h\left(t\right)\right)$ can arise also because the optimal strategy changes its prediction for the given *h*(*t*) (second and third terms in the bracket).

The change in time of the relative payoff *q*_*i*_ is computed as follows

$${\frac{\delta q_{i}}{\delta t}|}_{t}=\left[a_{i}^{2}\left(h\left(t-2\right)\right)\sum_{i=1}^{N}a_{i}^{*}\left(h\left(t-1\right)\right)\right]-\left[a_{i}^{1}\left(h\left(t-2\right)\right)\sum_{i=1}^{N}a_{i}^{*}\left(h\left(t-1\right)\right)\right]$$ (12)

Using $h\left(t\right)=\sum_{j=1}^{m}b\left(t-j+1\right)2^{j-1}$ and inserting (12) in (11) one obtains:

$$\begin{array}{l} {\frac{db}{dt}|}_{t+1}=\delta \left(\sum_{i=1}^{N}a_{i}^{*}\left(h\left(t\right)\right)\right)\sum_{i=1}^{N}\{\delta \left(q_{i}\left(h\left(t\right)\right)\right)\sum_{i=1}^{N}a_{i}^{*}\left(h\left(t-1\right)\right)\\ {}[{\text{a}}_{\text{i}}^{2}\left(\sum_{\text{j}=1}^{\text{m}}\text{b}\left(\text{t}-\text{j}-1\right)2^{\text{j}-1}-{\text{a}}_{\text{i}}^{1}\left(\sum_{\text{j}=1}^{\text{m}}\text{b}\left(\text{t}-\text{j}-1\right)2^{\text{j}-1}\right)\right)]\times \\ {}[a_{i}^{2}\left(\sum_{j=1}^{m}b\left(t-j+1\right)2^{j-1}-a_{i}^{1}\left(\sum_{j=1}^{m}b\left(t-j+1\right)2^{j-1}\right)\right)]+\\ \Theta (q_{i}\left(h\left(t\right)\right))\frac{\delta a_{i}^{2}\left(\sum_{j=1}^{m}b\left(t-j+1\right)2^{j-1}\right)}{\delta t}+\left[1-\Theta \left(q_{i}\left(h\left(t\right)\right)\right)\right]\frac{\delta a_{i}^{1}\left(\sum_{j=1}^{m}b\left(t-j+1\right)2^{j-1}\right)}{\delta t}\} \end{array}$$ (13)

If $\sum_{i=1}^{N}a_{i}^{*}\left(h\left(t-1\right)\right),\sum_{i=1}^{N}a_{i}^{*}\left(h\left(t-2\right)\right),\ldots ,\sum_{i=1}^{N}a_{i}^{*}\left(h\left(t-m\right)\right)$ have all the same sign, the right-hand-side of Eq. (13) becomes 0, thus proving that a constant bit *b(t)*, corresponding to either an exponential increase or decrease in price, is a Nash equilibrium.

## Phase Transitions in a Coarse-Grained Financial System

From the point of view of physics, the mathematical modelling of the agents’ decision making process described via the *b(t)* dynamics can be understood as a “magnetism”. Such magnetism is determined by the “spins” represented by the strategies, in which the interaction between different spins (the products of $\sum_{i=1}^{N}a_{i}^{*}\left(h\left(\cdot \right)\right)$ and$a_{i}^{j}$) are mixed with “free field” terms, namely the only terms without a payoff function (see Eq. (13)). Spin models are widely used to describe the dynamics of traders in financial markets by several researchers implementing statistical physics to inspect complex dynamics in finance. See for e.g., Refs [34, 35].

We would like to emphasize such a spins’ analogy of interaction in the description of a complex financial system, in which speculators and fundamentalists interact in a non-linear way with the objective to maximize their payoff function. It suggests another and more general perspective in order to understand the market dynamics based on the competition between $a_{i}^{j}$ and$a_{i}^{f}$, namely between technical analysis trading strategies and fundamental analysis trading strategies. Note that both technical and fundamental strategies can be active for different traders at the same time depending on the optimal strategies the agents possess at a given instant of time. Just like there is an interaction between spins in a magnet, there is an indirect interaction between market participants through their decision making, since the impact of one agents decision making can influence the future decision making of other agents through the price impact.

Taking this view, the financial market can be conceived as a thermodynamic system where its different states are characterized via the so-called free energy *F*, a concept in physics used to quantify the energy transferred by one system to another. The free energy plays a central role in physics, since its *minimum* determines how the state of the system will appear, and can be written as

F=E−TS (14)

with *E* the internal energy of the system, T the temperature and 𝒮 the entropy which one can think of as representing how much disorder there is in a given system. From the definition of *F* we can see that the state of a system is determined by a struggle between two different forces, one representing “order”, this is the *E* term, and the other term representing “disorder” given by the T𝒮 term.

A similar struggle of “forces” can be envisioned in the financial market between speculators and fundamentalists, where the general tendency to create either a positive/negative price trend corresponds to “order” state, whereas either the lack of consensus or the mean reversion to the fundamental price value will destroy such order thus moving the system into “disorder” state. Transitions and fluctuations in financial market are scrutinized in a number of recent contributions such as Ref. [36], in which transition between economic states is studied focusing on stock correlations, and in Ref. [37], where macroscopic “phase-flipping” phenomena are modeled in a dynamical network setting.

In terms of market macro-dynamics, such a reasoning stresses the importance to look at interactions among agents when trying to explain whether aggregate decisions are based on past price trend (speculative state), or on expected future dividends (fundamental state). The classical order (speculative) vs. disorder (fundamentalist) phase transition problem in physics, is turned over to fundamentalist (disorder) vs. speculative (order) mood (phase) transitions dilemma, that we propose to disentangle by picking up the general properties of the system through an adaptation of the well-known Ginzburg-Landau theory. It is with such a view that we observe the pen right at the borderline of falling and imagine a price fluctuation around its fundamental (fundamental state) until the pen breaks the symmetry by taking a clear direction as the price moves into bubble or anti-bubble mood (speculative state). The key point is to explain such dynamics through the “temperature” factor in Eq. (14) which moves the system from one mood to another.

### The Ginzburg-Landau theory

The central importance of the temperature factor can be seen in Eq. (14), in which we note that for temperature T = 0 the minimum of the energy *E* is therefore also the minimum of the free energy *F*. However as soon as T > 0, the finite temperature will introduce fluctuations in the system introducing thereby a non-zero contribution to the entropy *S*. The larger the temperature T the larger this tendency, until at a certain temperature *Tc* above which order has completely disappeared, and the system is in a disordered state. The GL theory explains how such a phase transition can be expressed in terms of an order parameter thus describing the general properties of the system without examining their microscopic properties. We do the same thing by exploring the macro-mechanisms of market transitions, while maintaining consistency in the micro-foundation of individual decision making.

Mathematically, the free energy *F* in Eq. (14) is assumed to depend on the temperature and the magnitude of the order parameter *m* upon which we can expand the series as follows:

$$F\left(T,m\right)=C+\alpha_{2}\left(T\right)m^{2}+\frac{1}{2}\alpha_{4}\left(T\right)m^{4}+\ldots$$ (15)

It should be noted that Eq. (15) does not contain odd terms (*m*, *m* ^3^, …) in the expansion due to a symmetry argument: there is no difference in the free energy for a spin up, respectively spin down system. As we will see below a similar financial symmetry exists expressed by the fact that there is no difference in the profit from a long, respectively short position.

Assume:

$$\begin{array}{l} \alpha_{2}\left(T\right)=a\left(T-Tc\right);a>0\\ \alpha_{4}\left(T\right)=b=constant>0. \end{array}$$ (16)

Taking furthermore the derivative in order to find its extreme, we end up with the equation for a minimum of *F*(T, *m*), hence determining the state of the system:

$$\frac{\partial F\left(T,m\right)}{\partial m}=2a\left(T-Tc\right)m+2bm^{3},$$ (17)

that has the following solutions:

$$\begin{array}{ll} 2a\left(T-Tc\right)m+2bm^{3}=0 & \\ 1)\;m\left(T\right)=0 & T\geq Tc\\ 2)\;m\left(T\right)=\pm \sqrt{\frac{a}{b}\left(Tc-T\right)} & T<Tc. \end{array}$$ (18)

Solution 1) describes a disordered state, while solution 2) is the solution describing an ordered state. The second solution determines the value of the critical exponent *β* when one expresses the order parameter as a function of the temperature near the critical point:

$$2\text{’})\;m\left(T\right)\to \Psi \left(T\right)={\left(Tc-T\right)}^{\beta }\;T<Tc$$ (19)

thus obtaining the so-called “mean field” or GL exponent of the transition β = 0.5. The GL theory offers through a power expansion a functional description of the free energy by integrating over the microscopic degrees of freedom, while constraining their average to *m*(T). By doing so, the phenomenological parameters assume an unknown functional dependence on the original microscopic parameters, as well as on the temperature, in this way accounting for the entropy of the short distance fluctuations lost in the coarse-graining (micro-to-macro) procedure.

### Mood transitions and the order parameter

The beauty of the GL theory is that one can describe phase transitions, without handling the microscopic description of interactions, simply via a power expansion of the order parameter. The structure of our market price dynamics lends itself to be treated in a similar vein, being conceivable as a complex system exhibiting an overall payoff function, as if it were the free energy of a thermodynamic system. Single particle movements are equivalent to the non-linear agent interactions (since one agent’s decision making can influence other agent’s decision making through price impact) that translate into a macro-dynamical environment in which:

- the ordered state corresponds to a speculative state, the price dynamics is going (up/down);
- the disordered state is instead corresponding to the fundamental state, in which the price moves randomly in a mean reversion process towards its fundamental value (Eq. (21)), thus destroying the trend in the ordered state.

To make the analogy with our discussion above (Eq. (14)), we introduce what we call the “Market Payoff” (*MP)* given by two terms in *MP* = *P−TS*.

- *P* is the total profit of the ordered state which for T = 0 corresponds to a continuous up/down trend of the market.
- *S* is an entropy term that destroys the ordered state, and T is the “temperature” which move the system from order to disorder and vice versa.

As discussed beforehand, the payoff of a strategy in the $-Game is equivalent to its profit, and agents use the same strategy over time in a Nash equilibrium. Therefore, the total profit *P* at time *t* for the system of traders can be written as:

$$P\left(t\right)=\sum_{i}\pi \left[a_{i}^{*}\left(t\right)\right]=\sum_{i=1}^{N}\sum_{l=1}^{N}a_{i}^{*}\left(t-1\right)a_{l}^{*}\left(t\right)\;\text{with}\;i\neq l,$$ (20)

where we note that the interactions among traders is, in a sense, “long-ranged”, since trader *l*’s action at time *t* has an impact on trader *i*’s profit from the action he/she took at time t—1.

In the GL theory the micro-to-macro thermodynamically description of the free energy needs consistency between particles micro- and macro-dynamics of the system, and this is obtained via the order parameter. Similar to the general case, for describing the macro-mechanisms of mood transitions we need an order parameter to expand the “Market Profit”, which also should maintain consistency in the micro-foundation of individual decision making.

As already observed, our trading setup is, in essence, the analog of a “magnetism” determined by single strategies chosen by the traders, who act as “atomic spins” moving in two possible directions, up (+1) or down (-1). As a result, the system as a whole moves between (and within) the two extremes all up (+*N*) and all down (-*N*), as in the physicist two-dimensional square-lattice Ising model, where the order parameter to describe phase transition is measured by the magnetism, which is just the averaged value of the spins. The Ising model is a model of ferromagnetism in which the energy *E = −J* ∑_<*i*, *j*>_ *s*_*i*_ *sj* with *s*_*i*_ and *s*_*j*_ representing the atomic “spins” of a material. The <>—notation in the summation indicates that the sum is to be taken over all nearest neighbors pair of spins. Each spin itself can be thought of as a mini magnet. In the two-dimensional Ising model the spin *s*_*i*_ = +1 if the spin is “up” and *s*_*i*_ = -1 if the spin is “down”. Taking the coupling strength between spins *J* positive, the minimum energy *E*_*min*_ of the system is simply given by either all spins up *s*_*i*_ ≡ +1), or all spins down (*s*_*i*_ ≡ -1). Although our trading setup is very similar to the Ising model, there is a major difference that refers to the “interaction” mechanism: in our framework, it is “long-ranged” (trader *l*’s action at time *t* has an impact on trader *i*’s profit from the action he/she took at time *t*—1), whereas the interaction is local (it only concerns nearest-neighbors) for the Ising model. Therefore, the order parameter we suggest to use in our market structure is the order imbalance:

$$ο\left(T\right)=\frac{1}{N}\sum_{i=1}^{N}a_{k}^{*}\left(t\right),$$ (21)

which gives us a simple way to understand phase transitions characterized by the following two extreme scenarios:

1. the case of $ο\left(T\right)=\frac{1}{N}\sum_{i=1}^{N}a_{k}^{*}\left(t\right)=\pm 1$ that corresponds to pure order in the system with all used strategies taking the same direction (buy or sell) and giving rise to a bubble or anti-bubble price path: this is the pure speculative state;
2. the case of $ο\left(T\right)=\frac{1}{N}\sum_{i=1}^{N}a_{k}^{*}\left(t\right)=0$ that corresponds, instead, to pure disorder in the system, with half of the population of used strategies (“spins”) taking one action and the remaining taking the opposite action, thus giving rise to a price path around its fundamental: this is the pure fundamentalist state.

### Financial symmetry

Applying now the GL idea and expanding *MP* in terms of *o(* T), one ends up with the very same conditions (Eq. (18)) to determine *o(* T), but since the objective of traders is to maximize their payoff function, the state of the system is determined by its maximum (maxima), instead of the minimum (minima), as it was the case for *MP(* T, *o)*.

Formally, by taking the derivative of *MP(* T, *o)* and solving for *o(* T) we obtain:

$$\begin{array}{l} \frac{\partial MP\left(T,ο\right)}{\partial ο}=2a\left(T-Tc\right)ο+2b{ο}^{3}=0\\ 1)\;ο\left(T\right)=0\;\;\;\;\;\;\;\;\;\;\;\;\;\;\;\;\;\;\;\;\;\;\;\;\;\;\;\;\;\;\;\;\;\;\;\;\;\;\;\;\;\;\;\;\;\;\;T\geq Tc\\ 2)\;ο\left(T\right)=\pm \sqrt{\frac{a}{b}\left(Tc-T\right)}\;\;\;\;\;\;\;\;\;\;\;\;\;\;\;\;\;\;\;T<Tc. \end{array}$$ (22)

Solution 1) gives *MP(* T, *o) ≡* 0 for o(T) = 0, corresponding to no price trend. This is the fundamental state in which randomness in price movements leads to no-arbitrage condition translating into a financial symmetry with a sort of mean-invariance within the system maintaining a general equilibrium in the market.

Solution 2) is with *MP(* T, *o) >* 0 for o(T) > 0 or o(T) < 0, corresponding to the speculative state with up or down price movements leading to a bubble or anti-bubble state breaking the financial symmetry and with agents getting positive profits by going long or short in the market.

Transitions from fundamental to speculative state are thus modulated through the “temperature” parameter, which play a central role in the macro-dynamic of the market. Indeed, financial symmetry is broken whenever the parameter crosses a critical transition point, *Tc*, which move the system from fundamental (with *MP(* T, *o) ≡* 0) to speculative state (*MP(* T, *o) >* 0.

**Fig 2:**
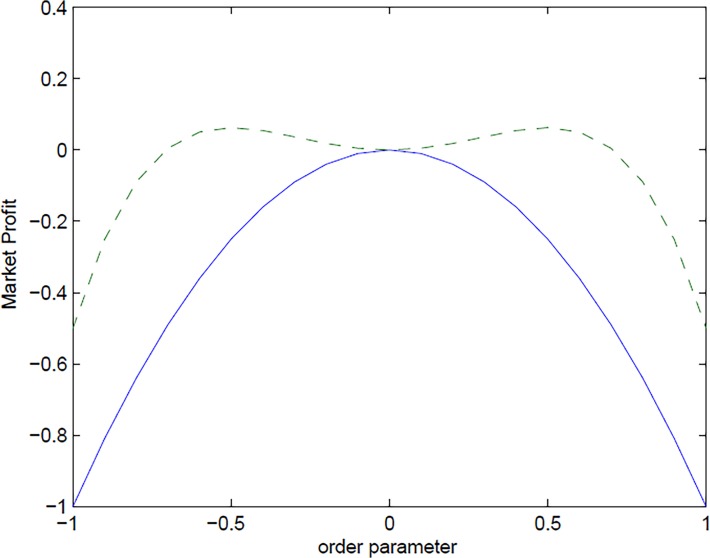
Market profit, financial symmetry and market temperature. The figure illustrates the “Market Profit” of *MP* expansion as a function of order parameter for two different “temperatures” corresponding to T ≥ *Tc* (solid blue line), and two T < *Tc* (dashed green line), respectively.

Fig. 2 illustrates the expansion of *MP* (y-axis) as a function of *o*(x-axis) for the two cases: i) the T ≥ *Tc* solution (i.e. the disordered state corresponding to no trend in the price path with *o(* T) = 0) can be seen as the maximum of the solid line, whereas the two T < Tc solutions (i.e. the ordered state corresponding to a specific trend in the price path with *o(* T) ≠ 0) can be found as the maxima of the dashed line. Note that when financial symmetry is “restored” in the market, the system is in equilibrium with no difference between profits from going long (buy) or short (sell) in the market (the maximum is *MP(* T, *o) ≡* 0). When instead financial symmetry is broken, due to a market temperature being less than the critical transition point (T < *Tc)*, the system finds its optima either from going long *o(* T) > 0) or from going short *o(* T) < 0) with the same maximum at (*MP(* T, *o)* > 0.

### The “market temperature”

One of the main implication of our GL-based theory of mood transitions in the market is the existence of a nontrivial transition from a “high temperature” symmetric state, where traders don’t create a trend over time, to a “low temperature” state, characterized by trend following with a definite trend in the price trajectories (up or down). We will define in the following a “temperature” linked to the randomness of the agent’s actions and suggest how it should be measured accordingly.

In our model setup, randomness enters the $-Game through the initial conditions in the assignments of the *s* strategies to the *N* traders in the game. In order to create a given strategy one has to assign randomly either a 0 or a 1 for each of the 2^*m*^ different price histories; therefore the total pool of strategies increases as $2^{2^{m}}$ versus *m*. However, many of these strategies are closely related: take for e.g. Table 1 changing just one of the 0’s to a 1, and note that this thereby creates a strategy which is highly correlated to the one seen in the table.

Refs. [11, 23] showed how to construct a small subset of size 2^*m*^ of independent strategies out of the total pool of $2^{2^{m}}$ strategies. As suggested in Refs. [24, 20], a qualitative understanding of this problem can be obtained by considering the parameter$\alpha =\frac{2^{m}}{N}$. Along the same line of reasoning, Ref. [25] pointed out, however, that the ratio $\alpha =\frac{2^{m}}{N\times s}$ seems more intuitive, since this quantity describes the ratio of the total number of relevant strategies to the total number of strategies held by the traders. Based on this intuition, and considering the presence of all relevant technical trading strategies (2^m^) together with *the* fundamental strategy, we then introduce the following measure for the market temperature in a speculative vs. fundamental moods transition financial system:

$$T=\frac{2^{m}+1}{N\times s},$$ (23)

where the “+1” in the numerator is because of the fundamental strategy together with the 2^m^ uncorrelated speculative strategies.

The relation of T to the fluctuations of the system becomes clear when one consider the fact that the variance of a small sample is larger than the variance of a large sample. This statement is called “the law of small numbers” in Psychology/Behavioral Finance and was introduced by Tversky and Kahneman (Refs. [26–28]). In our setup, we have a similar behavior: when the sample of strategies *s* held by the *N* traders is small with respect to the total pool of relevant strategies (reflecting in low denominator of T), this corresponds indeed to the large fluctuations, large temperature case. Vice versa a large sample of *s* strategies held by the *N* traders (increasing denominator of T), therefore corresponds to a small temperature case as seen from the definition of T.

## Simulation Study

### Experimental design

In our numerical experiment, we run Monte Carlo simulations based on the 5 parameters of our *in silico* financial system (*N*, s, λ, *D*(*t*)). A number of 200 simulations of the $-Game were run where each realization of the game were obtained for up to 200 × 2^m^ time steps. Numerically, we performed the experimental design in two main blocks of parameters: 1) *s* = 2; 2) s = 18. In this way we take into account both the case of small number of relevant strategies as well as the case of large number of strategies, since they substantially impact on our T parameter, as discussed in the previous section. For both the blocks, simulations were run starting from a stock price of 100 and varying parameters as follows:

- *N* = 11, 101. The two cases represent a thin and a large market.
- *m = 3*, *5*, *8*. The three values represent the number of past days traders use when forming their expectation and subsequently taking an action at time *t*; the parameter ranges from a short to a relatively long memory of the past;
- λ = 10, 100. We consider two levels of market liquidity which has an impact on price returns according to (2). The smaller the value of λ the larger the price impact of a given fixed value of the order imbalance.
- D(*t*) = 10, 20, 30, 40, 50, 60, 70, 80, 90, 100. We used ten possible scenarios for the expected dividends ranging from 10 to 100 percent of the dividend yield at the start of the simulation experiment (the price is set at 100). To simplify the model, the parameter is taken constant in time *t*.

The speculative and fundamental states are determined via the price path. Specifically, the system starts in a fundamental state with a *p*_*f*_(*t)* ≡ 100 and is defined to maintain financial symmetry whenever price fluctuations remain within a 50 percentage range (at maximum) of the fundamental price *p*_*f*_(*t)* converging to it at the end of the experiments. As discussed in the theoretical description of the model, people are assumed to trade shares of the company based on their expectations of future dividends, and the numerical experiment ends with the full price reflecting the expected dividends payout. This constraint is in line with those used in a large number of studies running experiments on stock trades (Refs. [29–33]).

**Fig 3:**
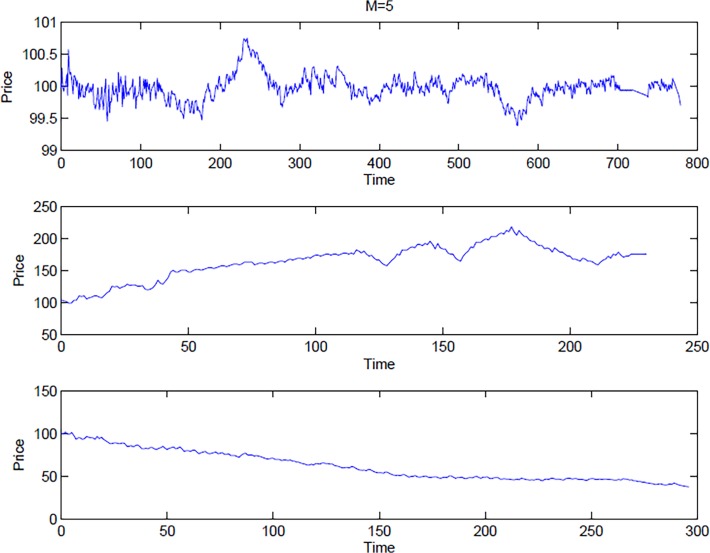
Three different market phases. The figure depicts three different examples corresponding to (from high to low): fundamental price behavior, speculative behavior in an increasing and decreasing market scenarios.

The speculative state is instead determined whenever *m* successive price changes had occurred. Fig. 3 shows three different results representing typical market behavior corresponding to fundamental price behavior, as well as speculative behavior in an increasing/decreasing market.

### Results

**Fig 4:**
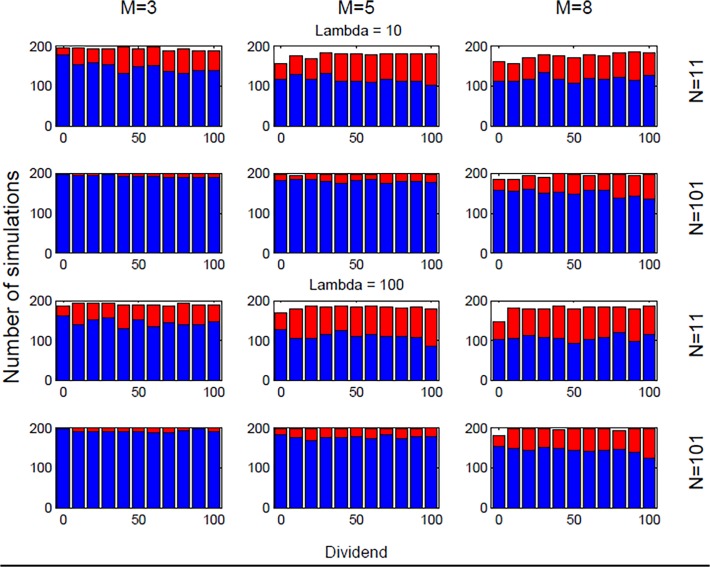
Fundamental vs. speculative market moods with *s* = 2. The figure reports histograms representing respectively speculative behavior (blue) or fundamentalist behavior (red) as outcomes in a setup of the $-Game for *s* = 2 with given parameter values of (*N*, *m*, *s*, λ, D(*t*)).

**Fig 5:**
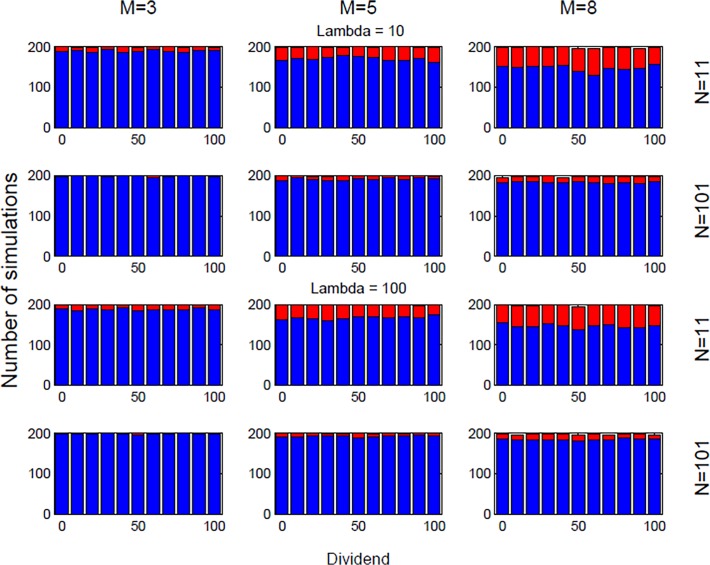
Fundamental vs. speculative market moods with s = 18. The figure reports histograms representing respectively speculative behavior (blue) or fundamentalist behavior (red) as outcomes in a setup of the $-Game for s = 18 with given parameter values of (*N*, *m*, *s*, λ, D(*t*)).

Figs. 4 and 5 show histograms representing respectively speculative behavior (blue) or fundamentalist behavior (red) as outcomes of our trading setup. The histograms in Fig. 4 represent simulations performed with s = 2 whereas the histograms in Fig. 5 were done for simulations with *s* = 18. We first notice the somewhat surprising fact that the dividends D(*t*) as well as the liquidity of the market λ, only seem to have a quite limited impact on the final state of the market. In particular, for the smallest *m* values (*m* = 3; 5), increasing dividends appear to have a somewhat stabilizing effect allowing for slightly more fundamental value states. The same stabilizing trend appears to be at play as one increase the liquidity of the market, but again, this tendency appears to be very weak. A much clearer tendency is seen with respect to increasing speculation when increasing the number of traders *N*, respectively, decreasing the amount of information *m* used in the decision making of the technical analysis trading strategies. A larger number of strategies *s* assigned to the traders is also seen to enhance speculation (compare Figs. 4 and 5).

Let us now explore how a qualitative behavior of this trading experiment can be predicted depending on our market temperature parameter T. The fact that T determines the outcome of the trading behavior can be easily seen by changing the nominator and denominator by the same factor, which then should lead to invariant behavior in terms of trading decisions. This means that for example the case of short-run memory and thin market (*m* = 3; *N* = 11) should give rise to a T = 0.8182 s^−1^ trading behavior. Such a trading behavior for a given λ and *s* should fall in between the medium-range memory and large market (*m* = 5; *N* = 101) (i.e. T = 0.3267 s^−1^) and long-range memory and large market (*m* = 8; *N* = 101) (i.e. T = 2.5446 s^−1^) cases. From Figs. 3 and 4 this is seen indeed to be the case. Similarly comparing Figs. 4 and 5 it is seen that increasing (/decreasing) *N* and decreasing (/increasing) *s* by the same amount leads to two systems behaving similarly in terms of investment profile (compare *N* = 101 rows in Fig. 4 to *N* = 11 rows in Fig. 5). Table 2 reports all values for T corresponding to small (*s* = 2) and high (*s* = 18) number of relevant strategies, respectively, showing how memory and number of traders impact on market temperature, and in turns on speculative vs. fundamental behaviors. Note that memory length seems to play a major role in moving the system between the two states of speculative, respectively fundamentalist behavior. The larger the *m* the higher the temperature, thus moving the aggregate market behavior towards a more fundamental investment oriented state. These results clearly underscore the importance of the parameter T when it comes to the understanding of the aggregate decision making in the model.

**Table 2:** T values.

*s* = 2	*N*	
***m***	***11***	***101***
***3***	0.409	0.045
***5***	1.500	0.163
***8***	11.682	1.272

***s* = 18**	***N***	
***m***	***11***	***101***
***3***	0.045	0.005
***5***	0.167	0.018
***8***	1.298	0.141

The table reports the values of the ratio the T ratio $\frac{2^{m}+1}{N\times s}$ run in our simulation experiment with *s* = 2 and *s* = 18. For these two scenarios the corresponding matrices derive by crossing *N* with *m* with *N* = 11, 101 and *m* = 3, 5, 8.

## Concluding Remarks

As discussed in Ref. [38], financial systems are complex adaptive system, in which the micro interactions translate into macro dynamics through bottom-up mechanisms, followed by top-down feedback between the macro and the micro. In this paper we introduced a novel theoretical framework to describe financial market macro-dynamics in which single agents interact in non-linear and complex ways. The classical order vs. disorder phase transition problem in physics is used here to explain the fundamentalist vs. speculative mood transitions in the markets, that we propose to disentangle through a Ginzburg-Landau-based power expansion. As observing the pen right at the borderline of falling, we imagine a price fluctuation around its fundamental until the pen breaks the symmetry by taking a clear direction as the price moves into bubble or anti-bubble mood.

Our Ginzburg-Landau-based theory of market mood dynamics explains a nontrivial transition from a “high temperature” symmetric state, with no price trends over time, to a “low temperature” state, with up or down price trends. The key parameter that moves the markets from one state to another is the “temperature parameter”, which we derive based on the randomness of the agent’s actions, the number and the memory length of traders.

In our simulation exercise we have shown how a qualitative understanding could be found depending on just our temperature parameter, thus bypassing the excessively complicated microscopic description of moods in the markets. Indeed, the temperature parameter modulates transitions between fundamental and speculative states without examining the micro-dynamics of single agents and their interaction impacts on the price path and connected market transitions.

The main message we offer in this paper is that through a phenomenological explanation of complex market dynamics, we are able to describe when markets as a whole are expected to change from fundamental to speculative states and vice versa, only by focusing on three variables: (1) the number of market traders (*N*), (2) their trading strategies (*s*) and (3) their past price movements memory (*m)*. Together these variables are assembled into the ratio $\frac{2^{m}+1}{N\times s}$ which is at the core of the complex market dynamics.

It should be noted that our method provides a framework to test the stability of a given market state and understand the influence of an external perturbation. One way to try to drive the market from one state to another would for example be to perturb (by e.g. forcing a large up/down price move) artificially the market when the agents have driven it into a fundamental state (or vice versa into the fundamental state when presently in a speculative state). Whether the agents would respond by driving the market further along the direction of the perturbation, or on the contrary, drive the market back to its unperturbed state is far from trivial. In Ref. [21] it was shown how considering decoupled strategies (see Ref. [21] for definition) was one way to get information about how the system of agents would reply to perturbations.

The proposed general framework can be used to describe the human decision making in a certain class of experiments performed in a trading laboratory, allowing us to predict the outcome of such type of trading experiments in terms of when to expect a fundamental versus a speculative state. Here we focused only on the theoretical description of the model, but in our future research agenda we will use our findings to the implementation of trading experiments performed in a trading laboratory.
